# Large vessel vasculitis combined with inflammatory bowel disease: epidemiology, shared pathogenesis, and therapeutic advances

**DOI:** 10.3389/fimmu.2026.1806045

**Published:** 2026-03-23

**Authors:** Minghao He, Yiping Yang, Yibing Hu

**Affiliations:** 1 Department of Cardiology, Affiliated Jinhua Hospital, Zhejiang University School of Medicine, Jinhua, Zhejiang, China; 2 Department of Gastroenterology, Affiliated Jinhua Hospital, Zhejiang University School of Medicine, Jinhua, Zhejiang, China

**Keywords:** giant cell arteritis, immunology, inflammatory bowel disease, Takayasu arteritis, vasculitis

## Abstract

Concomitant existence of large vessel vasculitis (LVV) and inflammatory bowel disease (IBD) is rare but has gained increasing clinical and research attention. LVV, comprising Takayasu arteritis (TAK) and giant cell arteritis (GCA), is characterized by granulomatous inflammation of the aorta and its branches. Accumulating evidence demonstrates a substantial overlap between LVV and IBD in epidemiological patterns, genetic susceptibility loci, immune cell subsets, and proinflammatory cytokine networks, supporting shared immunopathogenic mechanisms. IBD, including Crohn’s disease (CD) and ulcerative colitis (UC), is a chronic immune-mediated inflammatory disorder influenced by genetic predisposition and gut microbiota dyshomeostasis. Patients with concomitant LVV–IBD exhibit heterogeneous and often insidious clinical presentations, with a predominance in young women, frequent multisystem involvement, and significant diagnostic challenges. Multimodal assessment incorporating imaging, endoscopy, and laboratory biomarkers is essential for early detection, although unified diagnostic criteria and disease-specific biomarkers remain lacking. Management requires careful balancing of intestinal and vascular disease control. Current therapeutic approaches include glucocorticoids, conventional immunosuppressants, and biologic agents, with anti-tumor necrosis factor therapies and other targeted agents showing benefit in selected refractory cases. Future multicenter, large-scale prospective studies are needed to refine diagnostic strategies, optimize treatment algorithms, and further elucidate shared immune-inflammatory pathways to improve long-term outcomes and enable precision therapy.

## Introduction

Large-vessel vasculitis (LVV) primarily comprise giant cell arteritis (GCA) and Takayasu arteritis (TAK), both of which are characterized by granulomatous inflammation involving the aorta and its major branches (1). GCA predominantly affects women older than 50 years, whereas TAK commonly occurs in younger women. Although their clinical phenotypes differ, both conditions share key pathogenic features, including genetic susceptibility, immune-mediated vascular inflammation, and dysregulated cytokine signaling (2). Clinical manifestations range from non-specific systemic inflammatory symptoms to ischemic complications. Severe disease may result in irreparable vision loss, aortic aneurysm formation, arterial stenosis or occlusion, and major cardiovascular or cerebrovascular events (3). Glucocorticoids remain the cornerstone of treatment. Current recommendations support combination therapy with conventional immunosuppressive agents or biologics, such as tumor necrosis factor (TNF) inhibitors and tocilizumab. In addition, emerging therapies targeting the JAK/STAT pathway, IL-12/IL-23, and other immune pathways are under investigation. Optimal management requires an individualized approach guided by clinical practice guidelines, incorporating early diagnosis, vascular imaging, and multidisciplinary care to achieve sustained disease control and prevent vascular complications (4).

Inflammatory bowel disease (IBD) is a chronic, globally prevalent inflammatory disorder of the gastrointestinal tract, primarily encompassing Crohn’s disease (CD) and ulcerative colitis (UC) (5). The worldwide incidence of IBD has increased steadily over recent decades. Its pathogenesis is multifactorial, involving complex interactions among genetic predisposition, immune system dysregulation, impaired intestinal barrier function, and alterations in gut microbiota (5). Clinical manifestations are heterogeneous and commonly include abdominal pain, diarrhea, bloody stools, and weight loss. In addition, IBD is frequently associated with extraintestinal manifestations involving musculoskeletal, dermatologic, ocular, and vascular systems. Complications such as intestinal obstruction, fistula formation, abscesses, and colorectal cancer may occur, and severe or refractory disease may necessitate surgical intervention. Therapeutic strategies include 5-aminosalicylic acid, glucocorticoids, immunosuppressive agents, biologic therapies targeting TNF, integrins, or IL-12/23, and small-molecule agents such as JAK inhibitors. Contemporary management emphasizes early intervention, treat-to-target approaches, and individualized management based on disease phenotype and risk stratification (6, 7).

The coexistence of LVV and IBD is relatively rare in routine clinical practice (8, 9). Owing to overlapping inflammatory features, atypical presentations, and often insidious disease onset, the diagnosis of this comorbidity is frequently delayed or overlooked. These diagnostic challenges, coupled with the potentially severe consequences of delayed treatment, underscore the need for a comprehensive and systemic understanding of the association between LVV and IBD (10, 11). We conducted a comprehensive search for relevant studies published between January 1980 and December 2025 in PubMed, Web of Science, Embase, and Scopus databases. Articles meeting the following four criteria were eligible for inclusion: (1) confirmed coexistence of TAK or GCA with IBD (CD or UC); (2) diagnosis based on internationally recognized criteria; (3) study themes related to epidemiology, pathogenesis, or treatment. The exclusion criteria were as follows: (1) studies involving only a single disease or non-large-vessel vasculitis or non-IBD; (2) unclear diagnosis or incomplete information; (3) duplicate reports; (4) literature with insufficient relevance to the association between LVV and IBD.

This review aims to summarize the epidemiological characteristics of LVV complicated by IBD, explore potential shared pathogenic mechanisms, and synthesize current evidence regarding clinical manifestations, diagnostic approaches, and therapeutic approaches to improve clinical recognition and management of this complex comorbidity.

## Epidemiology

The coexistence of LVV and IBD is considered rare, and available evidence is largely derived from case reports and small observational studies. The earliest documented case was reported in 1976 by Yassinger et al., who described a 15-year-old patient with comorbid IBD and TAK (8). Subsequently, Kusunoki et al. summarized the clinical characteristics of 37 patients with CD complicated by TAK and reported that CD preceded or was diagnosed concurrently with TAK in 78% of cases; notably, four patients had undergone surgical treatment for CD (9). Based on theoretical estimates, the estimated incidence of CD comorbid with TAK has been suggested to be approximately one per ten billion. However, owing to the absence of large, multicenter population-based studies, reliable incidence data are lacking, and the true frequency is likely underestimated (9). Data from large IBD cohorts further support the rarity of this association. In a multinational cohort study including 5, 601 patients with IBD from Brazil and Israel, the prevalence of TAK was 0.12%. Similarly, a Japanese cohort study reported that only 0.21% of 1, 433 patients with UC were diagnosed with TAK (10, 11). In contrast, studies evaluating IBD prevalence within TAK cohorts have reported substantially higher rates. Several series have shown that IBD is present in up to 6.4% of patients with TAK, with some reports suggesting prevalences as high as 9.2% (12–14). These discrepancies may reflect differences in study design, cohort size, ethnic or geographic background, diagnostic vigilance, or referral patterns. In particular, variations in the specialty of the treating department may influence detection rates, as some specialties may be more familiar with LVV–IBD.

The association between TAK and IBD appears to be more frequent than that between GCA and IBD. Reports of GCA–IBD coexistence are relatively scarce, with only 12 cases described in earlier reports (15). TAK is considered the most common vasculitis subtype observed among patients with IBD (16). In the largest observational case–control study to date, involving patients from 29 European centers, the coexistence of GCA and IBD was indeed less frequent than that of TAK and IBD, with 39 cases of TAK–IBD compared with 12 cases of GCA–IBD (17). Within this cohort, CD predominated among patients with TAK–IBD, accounting for 67% (26/39) of cases, whereas UC was more common among patients with GCA–IBD, comprising 58% (7/12) of cases. However, population-based studies have yielded differing results. An Israeli nationwide cohort study reported a higher prevalence of CD among patients with GCA than among those with TAK (0.79% vs. 0.12%, *P* < 0.001), as well as a higher prevalence of UC in GCA compared with TAK (0.84% vs. 0.21%, *P* < 0.001); these associations remained significant after multivariable adjustment (18). Similarly, a Danish population-based cohort study demonstrated that the prevalence of GCA was significantly higher in patients with IBD overall and in those with UC specifically, compared with controls (odds ratio [OR] 1.6 for both), whereas no significant association was observed in patients with CD (19). These discrepancies across studies may reflect regional variations in the relative incidence of UC and CD within IBD cohorts, as well as a potential phenotypic predilection for CD in TAK–IBD and for UC in GCA–IBD. Larger-scale studies are needed to further clarify the true magnitude and direction of these associations.

With respect to temporal sequence, available evidence suggests that LVV usually develops after the onset of IBD. The median interval between IBD and vasculitis diagnosis has been reported as 1 year (interquartile range [IQR] 1–7) for patients with TAK–IBD and 8.6 years (IQR 1–17.7) for those with GCA–IBD (17). Notably, LVV is frequently diagnosed during periods of IBD remission. This study found that LVV developed in IBD patients who were already receiving immunosuppressive therapy, including high-dose glucocorticoids (36%), azathioprine (25%), and TNF-α inhibitors (29%). One possible explanation for this observation is that patients with LVV–IBD exhibit relative refractoriness to conventional immunosuppressive regimens, necessitating more intensive treatment regimens. Alternatively, LVV may represent a paradoxical immune-mediated reaction associated with TNF-α inhibitor therapy, as vasculitis is a rare but well-recognized adverse event linked to these agents (20). Despite the predominance of IBD preceding LVV, reverse or concurrent disease onset has also been reported. For instance, a recent case from India described a middle-aged man who initially presented with heart failure and aortic regurgitation, in whom computed tomography angiography (CTA) confirmed TAK. During hospitalization, the patient developed hematochezia, and subsequent colonoscopy and biopsy established a diagnosis of CD. Combined treatment with glucocorticoids, mesalamine, and azathioprine resulted in effective control of both cardiovascular and gastrointestinal manifestations, highlighting the systemic and heterogeneous nature of autoimmune diseases (21).

Regarding age at diagnosis, patients with IBD–TAK are significantly younger at the time of TAK diagnosis than patients with isolated TAK (27 vs. 37 years, *P* < 0.001). In contrast, no significant difference in age at diagnosis has been observed between patients with GCA–IBD and those with isolated GCA (68 vs. 72 years, *P* = 0.086) (17). Further insight is provided by a large cross-sectional study based on an Israeli nationwide health registry, which demonstrated an inverse association between age and the strength of GCA–IBD relationship. Specifically, the association was strongest among middle-aged and older patients aged 50–69 years (OR 8.13) and weaker among elderly patients aged 70–85 years (OR 3.81). These findings suggest that clinicians should maintain a heightened index of suspicion for comorbid IBD in middle-aged and older patients with GCA, particularly when vasculitic manifestations occur at a relatively earlier stage (18).

**Figure 1:**
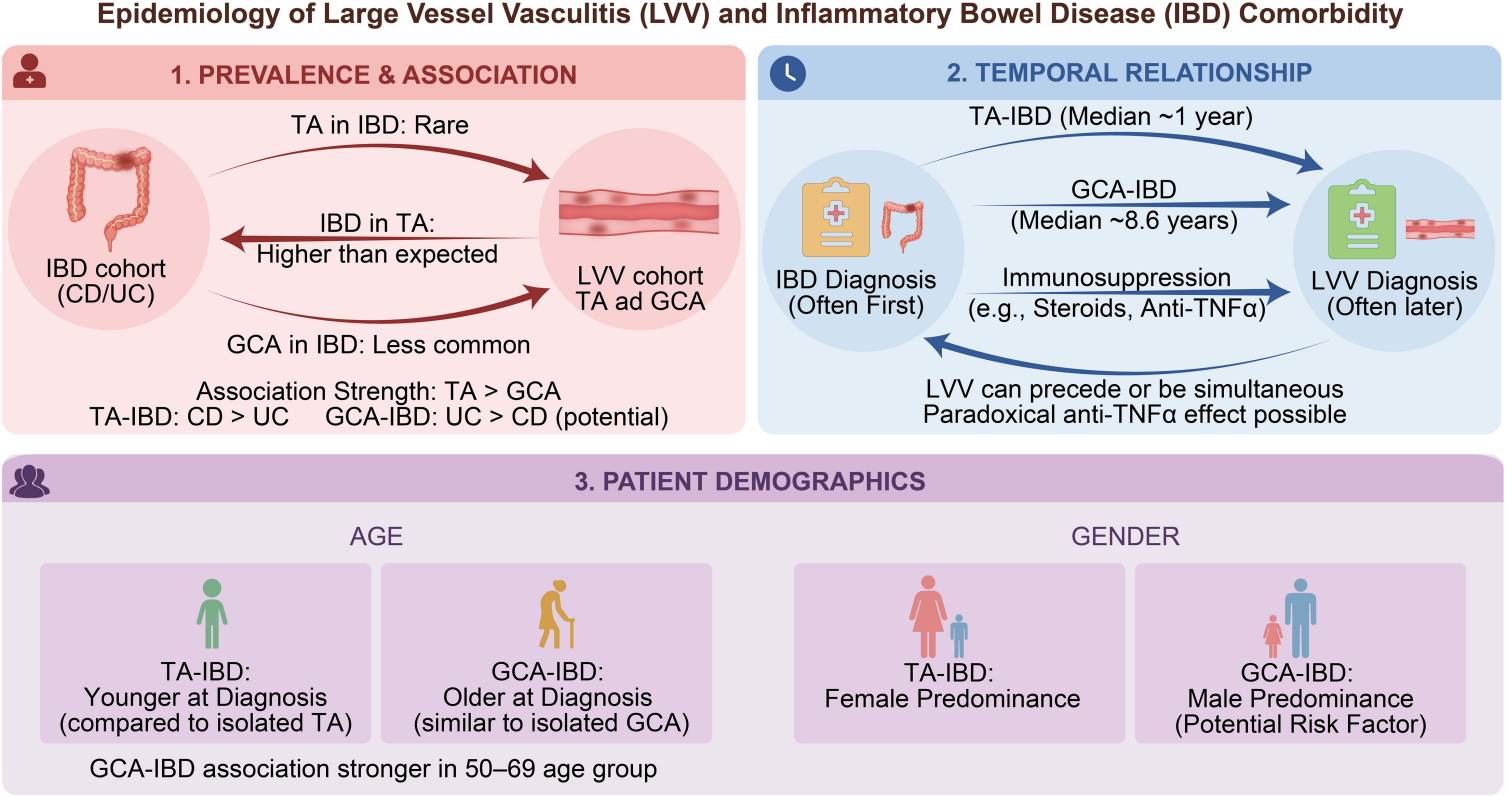
Epidemiology of comorbid large vessel vasculitis (LVV) and inflammatory bowel disease (IBD).

Sex-related differences have also been observed. Young women predominate among patients with IBD–TAK, consistent with the known female predominance of TAK. Among nine reported cases of IBD–TAK, eight occurred in women (16, 22). In contrast, male patients appear to be overrepresented among those with GCA–IBD. In the study by Bekele et al., men accounted for 66% (4/6) of patients with GCA complicated by IBD, while women accounted for 33% (2/6). When these data were combined with a systematic literature review, the male predominance persisted (75% vs. 25%) (15). Given that isolated GCA typically shows a female predominance, male sex may represent a distinguishing clinical characteristic or potential risk factor for GCA comorbid with IBD (**Figure 1**).

## Pathogenesis

Given the substantial overlap in immunoinflammatory features between IBD and LVV, it has been proposed that these conditions may represent distinct organ-specific manifestations of a shared inflammatory process affecting the intestine and the vasculature (23). The mechanisms underlying their coexistence are likely multifactorial and involve genetic susceptibility, immune dysregulation, infectious or environmental triggers, and overlapping inflammatory cytokine networks. However, the precise biological relationship between IBD and LVV has not yet been fully elucidated.

Genetic predisposition, particularly within the human leukocyte antigen (HLA) system, appears to play a central role in disease overlap. Compared with patients with isolated TAK, those with IBD–TAK show significantly higher frequencies of HLA class I alleles, including HLA-B*52:01 and HLA-C*12:02, as well as class II alleles such as HLA-DRB1*15:02, HLA-DQA1*01:03, DQB1*06:01, and DPB1*09:01 (10). Another study reported a markedly higher prevalence of HLA-B*52:01 in patients with UC–TAK compared with those with isolated TAK (92.6% vs. 50.7%; OR 12.14 [95% CI 2.96–107.23]). Furthermore, UC and TAK share common non-HLA genetic susceptibility loci, including the IL12B rs6871626 allele, implicating shared cytokine signaling pathways in disease pathogenesis (14). Additionally, the HLA-DRB1*04 allele has been identified as a shared genetic risk factor for both GCA and CD (24). Therefore, heightened clinical vigilance and consideration of screening for coexisting disease may be warranted in LVV or IBD populations carrying high-risk alleles, particularly HLA-B*52.

In addition, a case report described the first patient with X-linked inhibitor of apoptosis protein (XIAP) deficiency complicated by Crohn’s disease–like ileocolitis and TAK. The patient was a 10-year-old boy initially diagnosed with CD who showed a poor response to conventional therapy and required prolonged corticosteroid treatment and parenteral nutrition. At 15 years of age, he developed IgA vasculitis, and by 17 years, his disease progressed to refractory TAK. Whole-exome sequencing identified a novel splice-acceptor site mutation in the XIAP gene (c.1057-1G>A), resulting in markedly reduced XIAP mRNA expression. At 20 years of age, the patient underwent allogeneic hematopoietic stem cell transplantation (allo-HSCT), after which both enteritis and TAK achieved sustained clinical remission. This case highlights the role of monogenic immune dysregulation in severe, treatment-refractory inflammatory disease involving both the intestinal tract and the vasculature, and suggests that allo-HSCT may represent a curative option in selected patients (25).

Accumulating evidence indicates that T-cell–mediated immune responses play a central role in the pathogenesis of both IBD and LVV. In particular, Th17 and Th1 cell subsets have been implicated in both conditions (26). Activation of these pathways leads to the production of pro-inflammatory cytokines, including TNF-α, IL-1, and IL-12. Among these mediators, TNF-α is critical for granuloma formation, a histopathologic feature observed in both CD and TAK, supporting a shared mechanism of cell-mediated immune injury and providing a biological rationale for the use of anti-TNF-α therapy (27, 28). IL-6 also plays a role in vascular inflammation in TAK and is markedly elevated in the serum and colonic mucosa of patients with UC, where its levels correlate closely with disease activity. These observations suggest that IL-6 may represent a relevant therapeutic target for UC–TAK (29, 30). In addition, emerging data suggest that the association between UC and GCA may involve helper CD4 T cells (Th9 cells) and their signature cytokine IL-9, as increased IL-9 expression has been reported in both diseases. However, the cellular sources of IL-9 and its precise role in disease initiation and progression remain incompletely defined and warrant further investigation (8).

Although anti-TNF-α agents, such as infliximab, are effective in many patients with LVV–IBD, their use has paradoxically been associated with the development of TAK in some patients with pre−existing CD. Form a mechanistic perspective, TNF-α blockade may disrupt immune regulatory networks, leading to T-cell imbalance and the induction or unmasking of vasculitic processes, a phenomenon commonly referred to as a “paradoxical effect” (31). An alternative hypothesis proposes that the emergence of TAK may reflect subtherapeutic drug exposure rather than a direct drug-induced adverse effect. Inadequate serum concentrations of infliximab may result in insufficient suppression of shared Th1-driven inflammatory pathways implicated in both LVV and IBD, thereby allowing vascular inflammation to evolve despite treatment. Some reports have proposed Mycobacterium tuberculosis infection as a potential common trigger for LVV and IBD, though supporting evidence remains limited (32).

Recent evidence has identified antibodies against integrin αvβ6 as a highly specific serological marker in patients with UC. In one study, 87.5% of patients with TAK comorbid with UC tested positive for anti-αvβ6 antibodies, compared with only 3.18% of patients with TAK without UC (OR 121, P = 7.46 × 10^−8^). These antibodies are thought to contribute to intestinal mucosal injury, and the observed association appears to be independent of shared genetic susceptibility, including HLA-B*52. Thus, anti-αvβ6 antibodies may serve as a UC-specific biomarker, retaining high specificity even in the presence of concomitant TAK (33).

In contrast, isolated case reports have described alternative temporal patterns that argue against a direct pathogenic link in all patients. One such report involved a 24-year-old Caucasian female medical student who was incidentally diagnosed with clinically quiescent TAK after detection of bilateral carotid bruits. Nine months later, she developed abdominal pain, vomiting, and abdominal distension, and CD was subsequently diagnosed based on radiological findings and surgical pathology. In this case, CD developed after several years of quiescent TAK. Therefore, some investigators have proposed that the co-existence of TAK and CD may, in certain patients, reflect overlapping demographic susceptibility, particularly among young women, rather than a direct causal or mechanistic relationship between the two conditions (34).

**Figure 2:**
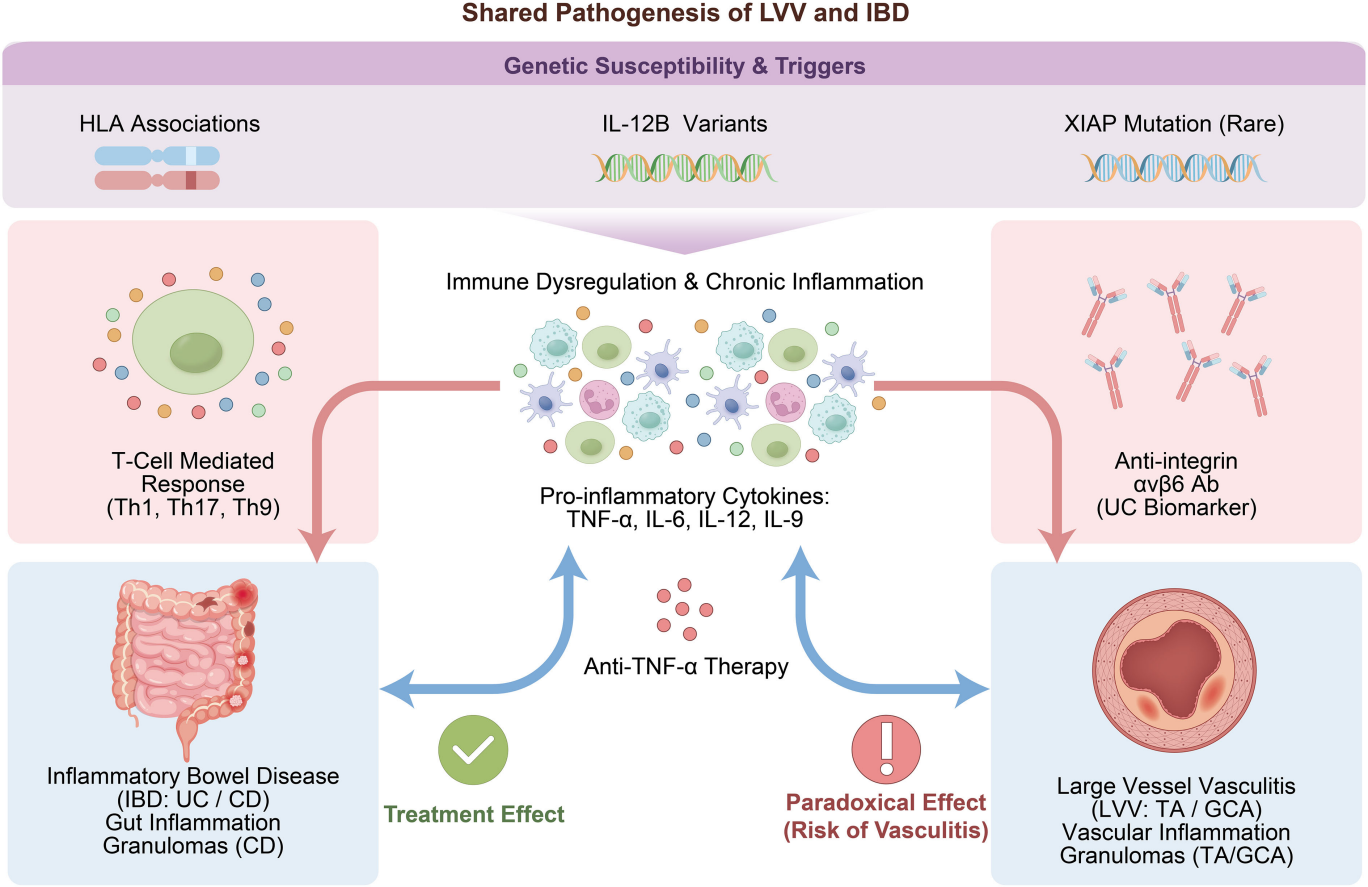
Shared pathogenesis of LVV and IBD.

Overall, available evidence supports a nonrandom association between IBD and LVV, particularly TAK. Although the coexistence of these conditions remains uncommon, epidemiologic and immunologic data suggest shared pathogenic pathways in at least a subset of patients. Larger, multicenter prospective studies are required to further delineate the underlying mechanisms and to refine diagnostic and therapeutic strategies for this complex comorbidity (**Figure 2**).

## Clinical manifestations

The early clinical presentation of concomitant LVV and IBD is often nonspecific. Patients may present with constitutional symptoms such as fever, headache, and fatigue, frequently in the absence of characteristic laboratory abnormalities, and some individuals may remain asymptomatic. One report described a female patient with refractory TAK who exhibited persistently elevated acute-phase reactants and progressive vascular lesions despite treatment with glucocorticoids and multiple immunosuppressive agents. Subsequent colonoscopic evaluation revealed active but asymptomatic CD, and initiation of treatment with adalimumab resulted in clinical improvement. This case underscores the importance of considering occult IBD in patients with refractory TAK (35). Inflammatory markers, including erythrocyte sedimentation rate (ESR) and C-reactive protein (CRP), may aid in diagnosis. As the disease progresses, clinical manifestations become more dependent on the distribution and severity of vascular involvement. Typical features include limb claudication, pulselessness, hypertension due to renal artery stenosis, or ischemic symptoms related to involvement of major arterial branches (31). Gastrointestinal ischemia represents a rare but severe manifestation. For example, a reported case described a 27-year-old man with a 10-year history of UC who presented with acute abdominal pain and was found to have acute ischemic colitis secondary to TAK. Imaging revealed edema of the ascending colon, ascites, and thickening of the aortic wall with mild stenosis of the superior mesenteric artery. This case highlights that vasculitis should be considered in patients with UC who present with atypical or severe colitis, and that early recognition may prevent catastrophic outcomes (36).

Comparative analyses further indicate that systemic and vascular manifestations are more prominent in patients with concomitant disease. Compared with patients with isolated LVV, those with IBD–TAK more frequently present with upper limb claudication (36% vs. 12%, *P* = 0.006) and abdominal pain (26% vs. 9.8%, *P* = 0.018). In contrast, patients with GCA–IBD more commonly exhibit vascular bruits (25% vs. 0%, *P* = 0.003) and arthralgia (50% vs. 15%, *P* = 0.017) (17).

LVV–IBD may present with diverse comorbidities and atypical, multisystem manifestations. Recently, the first reported case of concomitant UC, ankylosing spondylitis (AS), TAK, and asymptomatic thyroiditis highlighted the systemic nature of immune dysregulation in this setting. In that case, the patient sequentially developed UC, AS, and TAK, complicated by cerebellar stroke and thyroiditis, and ultimately achieved significant clinical improvement with infliximab therapy. This report underscores the potential for extensive multisystem involvement in LVV–IBD and the need for comprehensive evaluation (37). Additional reports describe severe and unexpected neurological complications. Amirreza et al. reported a 44-year-old woman with concomitant UC and TAK who presented with acute ischemic stroke, unilateral limb weakness, and visual impairment without preceding systemic symptoms. Imaging demonstrated occlusion of the basilar artery and right posterior cerebral artery, along with diffuse aortic branch vessel wall thickening consistent with TAK. Colonoscopic findings of friable, hyperemic mucosa with cryptitis and crypt abscesses confirmed UC. The patient also had hepatocellular adenoma, primary sclerosing cholangitis, and lymphadenopathy, underscoring the importance of multidisciplinary collaboration in managing such complex cases (38).

Hypercoagulable states may further compound vascular risk. Tarun et al. described reduced antithrombin III activity (54%) in a female patient with CD-TAK, resulting in a prothrombotic state that, together with large-vessel stenosis and thrombosis, markedly increased the risk of ischemic stroke (39). Even more rarely, antiphospholipid syndrome has been implicated. One report described a female patient diagnosed with CD at 16 years of age following perforated appendicitis, who later developed stricturing disease requiring surgery. At 20 years of age, during recovery after cholecystectomy, she experienced acute pulmonary thromboembolism attributed to double-positive antiphospholipid syndrome. Subsequently, at 26 years of age, she developed symptoms of large-vessel inflammation, including tinnitus, headache, and bilateral carotid pain, and was ultimately diagnosed with TAK (40).

Autoimmune clustering has also been observed. Another case involved a 31-year-old woman who was diagnosed with TAK at 16 years of age, who later developed Hashimoto thyroiditis at 18 years, followed by Sjögren syndrome in adulthood. Persistent hematochezia and periumbilical pain eventually prompted colonoscopic evaluation, which revealed extensive mucosal inflammation, and histopathological findings consistent with UC, leading to the diagnosis of concomitant IBD (41). Collectively, these cases illustrate that LVV–IBD may manifest as a complex, multisystem inflammatory disorder with insidious onset and potentially severe complications. Heightened clinical vigilance and in-depth investigation are needed to better characterize these rare but clinically significant presentations.

From an imaging standpoint, patients with GCA–IBD more frequently exhibit arterial wall thickening or luminal stenosis than patients with GCA without IBD (75% vs. 30%, *P* = 0.044). In addition, a higher tendency toward gastrointestinal arterial involvement has been observed in the GCA–IBD group (20% vs. 0%, *P* = 0.06) (17).

**Figure 3:**
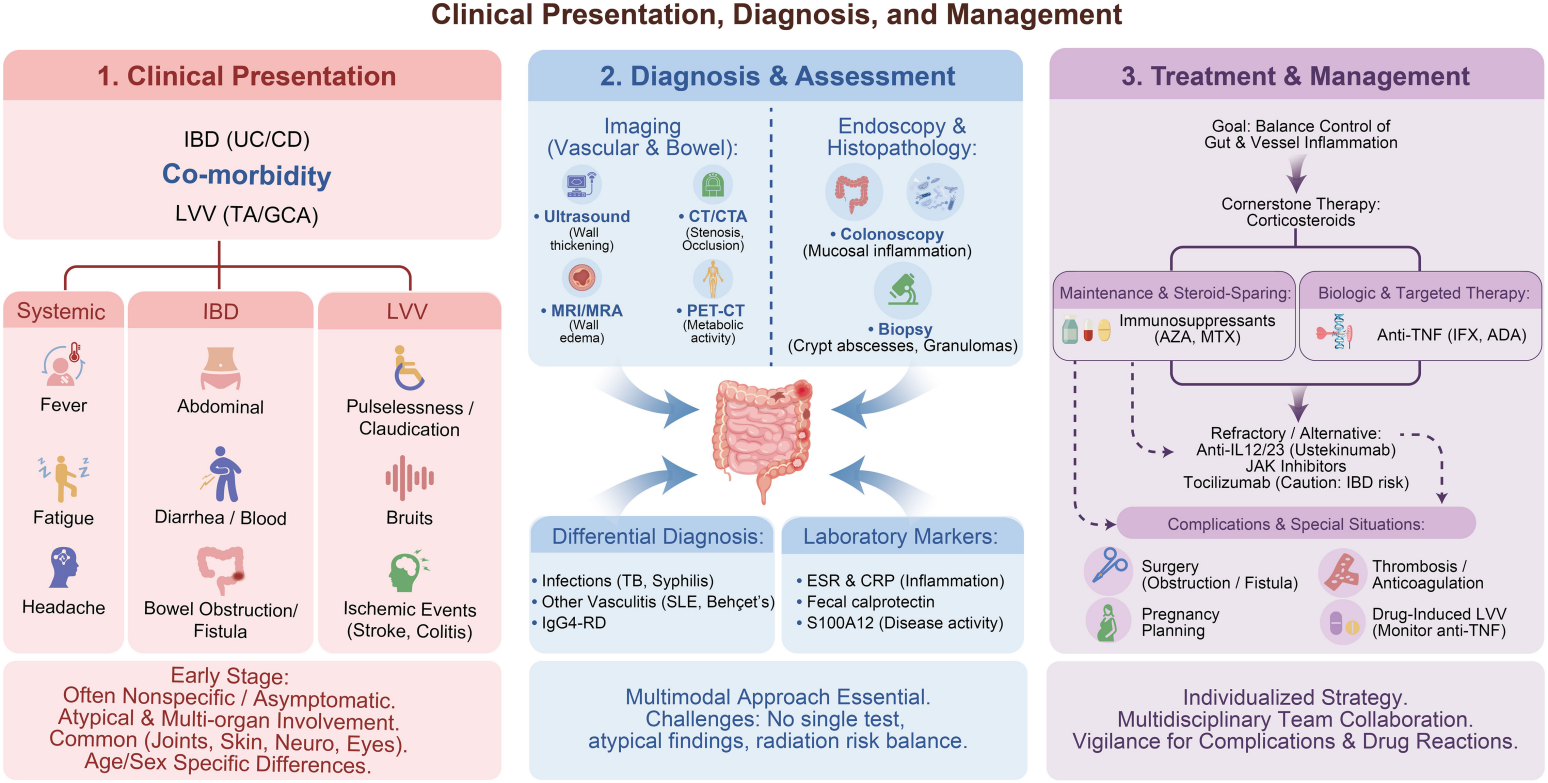
Clinical presentation, diagnosis, and management of LVV–IBD.

In summary, IBD complicated by LVV exhibits distinct differences in age, sex, and affected systems. The disease course is often insidious and may involve multiple organs, with imaging features that can aid in early detection. Early recognition and multidisciplinary collaborative assessment are crucial for improving prognosis in such cases (**Figure 3**).

## Diagnosis and assessment

Currently, the diagnosis of LVV–IBD continues to rely on established diagnostic criteria for each condition. In adults, TAK and GCA are diagnosed according to the 2022 American College of Rheumatology and European Alliance of Associations for Rheumatology (EULAR) classification criteria, whereas pediatric TAK is diagnosed using the 2010 EULAR/PRINTO/PRES criteria (1). The diagnosis of IBD follows the 2006 Montreal classification, incorporating endoscopic, radiologic, and histopathologic findings (5, 42). Important differential diagnoses include tuberculosis, syphilis, systemic lupus erythematosus, IgG4-related disease, and Behçet’s disease. No single diagnostic modality is sufficient to comprehensively evaluate large-vessel vasculitis; therefore, multimodal imaging plays a central role in diagnosis and disease assessment. Commonly used techniques include Doppler ultrasound, CTA, magnetic resonance angiography (MRA), positron emission tomography–computed tomography (PET-CT), and digital subtraction angiography (DSA), with Doppler ultrasound being the most frequently applied initial modality (17).

Each imaging modality has its own advantages and limitations. MRA provides high-resolution information on arterial wall thickness, edema, and delayed gadolinium enhancement without ionizing radiation. CTA offers diagnostic accuracy comparable to MRA for evaluating luminal changes, but it carries a risk of radiation exposure (43). These imaging techniques allow assessment of the degree of arterial stenosis, calcification, and occlusion (44–46). In recent years, the diagnostic importance of PET-CT has gained increasing attention for its ability to detect metabolically active vascular inflammation. Increased tracer uptake in the aorta and its major proximal branches enables early detection of vasculitic lesions and provides complementary information for assessing disease activity (47). However, PET-CT involves higher radiation exposure and is not widely available.

A reported case involving a 15-year-old girl with CD demonstrated the value of this multimodal approach. Initial chest CT and CTA revealed bilateral pleural effusions and arterial wall thickening of the left subclavian and pulmonary arteries. Subsequent 18F-FDG PET/CT showed increased metabolic activity in the aortic arch, left subclavian artery, and bilateral pulmonary arteries, consistent with active vasculitis. After three months of therapy, follow-up MRA demonstrated persistent arterial wall thickening with delayed gadolinium enhancement, confirming ongoing inflammatory activity (48).

DSA, although capable of delineating luminal contour abnormalities, is limited by its invasive nature and inability to evaluate arterial wall inflammation. Consequently, it has gradually been replaced by CTA or MRA (43, 49). In contrast, ultrasound plays an important role because of its accessibility, repeatability, and sensitivity in detecting arterial wall thickening (43). Typical ultrasonographic findings in TA include diffuse thickening of the carotid intima–media complex. As a noninvasive and radiation-free modality, ultrasound is well-suited for screening, particularly in young patients or those with atypical symptoms. It is often used in combination with CT, MRI, and PET-CT to improve diagnostic accuracy and enable early detection (50). An illustrative case involved a 29-year-old woman with UC who presented with right-sided neck pain and was ultimately diagnosed with TAK. T2-weighted MRI demonstrated concentric wall thickening of the right common and internal carotid arteries, with hyperintensity of the intima–media complex and hypointensity of the adventitia. Color Doppler sonography revealed homogeneous hypoechoic wall thickening (the “macaroni sign”) and abnormal arteriovenous mixed flow. MRA further identified early arterial flow within the right cervical venous plexus, consistent with a rare TAK-associated cervical arteriovenous fistula. These imaging features, including concentric wall thickening, absence of intramural hematoma, involvement of surrounding vascular structures, and AV shunting, help distinguish TAK from arterial dissection. This represents the first reported case of a cervical venous plexus arteriovenous fistula associated with TAK (51, 52). Although vascular biopsy may provide histopathological confirmation, it is technically challenging, carries procedural risk, and is often impractical in large-vessel disease (52).

Endoscopic assessments in patients with LVV-IBD should recognize that intestinal involvement may display atypical patterns. A retrospective analysis of endoscopic findings in eight patients with IBD complicated by TAK demonstrated that 87.5% (7/8) exhibited discontinuous and focal mucosal inflammation, while only one patient showed continuous rectal inflammation resembling classic UC. These endoscopic patterns differ from those typically observed in isolated UC or CD and may therefore contribute to diagnostic delay or misclassification. Accordingly, in patients with TAK who present with gastrointestinal bleeding or positive fecal occult blood tests, the possibility of concomitant IBD should be considered, with particular attention to atypical endoscopic findings (53). One illustrative case involved an 18-year-old woman who initially exhibited discontinuous colonic inflammation suggestive of CD, which gradually evolved over 10 years into continuous inflammation resembling UC. During treatment with infliximab, she was subsequently diagnosed with TAK, suggesting that TAK should be considered in patients with atypical patterns of colonic inflammation accompanied by fever of unknown origin (54).

With respect to laboratory assessment, elevations in ESR and CRP are commonly observed during active phases of LVV–IBD but lack disease specificity. Fecal calprotectin provides a useful marker for assessing intestinal inflammation in this context. Moreover, a study measuring fecal S100A12 levels in 30 patients with TAK and 14 controls demonstrated, for the first time, its potential clinical relevance in TAK. Fecal S100A12 levels were significantly higher in patients with TAK than in controls and showed a positive correlation with overall disease activity. Notably, patients with TA who reported abdominal symptoms exhibited particularly elevated fecal S100A12 levels. These findings suggest that fecal S100A12 may serve as a convenient and noninvasive biomarker for assessing disease activity in TA and for identifying potential concomitant intestinal inflammation (55).

Overall, accurate diagnosis of LVV–IBD requires a comprehensive, multimodal assessment integrating imaging, endoscopy, and laboratory biomarkers. Owing to the heterogeneity of clinical presentations and the absence of disease-specific diagnostic tests, early recognition remains challenging. There is a pressing need for large, multicenter studies to establish standardized diagnostic protocols that incorporate clinical features, emerging biomarkers, and advanced imaging techniques, thereby improving diagnostic accuracy and optimizing patient management (**Figure 3**).

## Treatment and management

The treatment of LVV–IBD presents substantial challenges. These challenges arise primarily from two considerations. First, LVV is frequently diagnosed after the onset of IBD, necessitating reassessment and potential modification of ongoing intestinal-directed therapy at the time of LVV diagnosis. Second, clinicians must carefully balance the efficacy and safety of therapies when simultaneously managing two chronic inflammatory conditions with overlapping but not identical treatment paradigms. Glucocorticoids remain the cornerstone of initial therapy for both conditions and can rapidly alleviate systemic symptoms and suppress inflammatory activity. In patients who are refractory to glucocorticoids, experience relapse, or encounter difficulty with dose tapering, adjunctive immunosuppressive agents such as azathioprine or methotrexate are commonly employed, particularly to maintain remission and reduce steroid dependence. Biologic therapies, including TNF inhibitors, may also be considered in selected patients. Surgical intervention is reserved for patients with severe intestinal obstruction, fistula formation, or vascular complications (9).

Several studies have reported the use of TNF inhibitors in patients with IBD–TAK who were refractory to conventional therapy. In one series involving nine such patients, anti-TNF monoclonal antibodies were associated with significant clinical improvement and reduced glucocorticoid requirements, suggesting potential efficacy in refractory disease (22). However, therapeutic responses have been heterogeneous, and in some patients, TAK activity failed to improve with a continuous progression of vascular lesions despite treatment. Anti-TNF therapy may also represent an option for women with concomitant disease who wish to conceive. One reported case described a 33-year-old woman with concomitant TAK, double-positive antiphospholipid syndrome, and CD who completed two spontaneous pregnancies and deliveries while receiving infliximab in combination with azathioprine and anticoagulation, without the use of glucocorticoids (40). Despite these potential benefits, the risk of infection associated with anti-TNF therapy warrants careful consideration. A reported case involved a 38-year-old man with a 15-year history of CD who received sequential treatment with infliximab and adalimumab but required discontinuation of both agents because of severe infectious complications, including sepsis and graft thrombosis. Ultimately, disease stability was achieved only with glucocorticoids and azathioprine. This case highlights that although anti-TNF therapy may be effective in selected patients with concomitant CD and TAK, the risk of serious infection may be substantial and necessitates cautious patient selection and close monitoring (56).

The development of LVV during anti-TNF therapy warrants special attention as drug-related immune phenomena must be considered and treatment discontinuation may be necessary. A recent report described a 16-year-old adolescent with CD who developed clinical features of TAK, including left-sided limb pain and loss of pulse in the left arm, while intestinal disease remained well controlled with infliximab. Following discontinuation of infliximab and initiation of prednisone combined with methotrexate, inflammatory markers normalized within three months. Similar cases have been reported in the literature (31, 48). Such events are not limited to CD. One case involved a 22-year-old woman with UC who developed new-onset TAK during infliximab therapy. Although her UC symptoms improved and prednisolone was successfully tapered, she developed neck pain and elevated CRP levels after the fourth infliximab infusion. Subsequent ultrasonography and CTA confirmed TAK. Notably, the patient carried the HLA-A24 and HLA-B52 haplotypes. Given that infliximab IFX is commonly used to treat refractory TAK, this case raises the possibility that anti-TNF therapy may trigger vasculitis in genetically susceptible individuals or that subclinical TAK becomes clinically apparent as drug efficacy wanes (57). In patients with LVV-IBD who show inadequate response to anti-TNF therapy, insufficient drug exposure should also be considered. Naoki et al. reported a patient with concomitant TAK and CD who experienced increased vasculitic activity during infliximab maintenance therapy, with disease control restored after dose escalation and shortening of the dosing interval. Although TNF-α is a key mediator in the pathogenesis of both diseases, the mechanisms underlying TAK flare or new-onset TAK during anti-TNF treatment remain incompletely understood (22). Collectively, these observations indicate that during anti-TNF therapy, particularly in patients with CD, clinicians should remain vigilant for the emergence or progression of TAK and consider both paradoxical immune reactions and inadequate dosing. Moreover, TAK has also been reported to become clinically apparent after discontinuation of anti-TNF therapy in patients with CD. Long-term, prospective studies are therefore needed to clarify the complex relationship between anti-TNF treatment and TAK with respect to both efficacy and safety (58).

Some experts propose that when LVV develops in patients with IBD who are already receiving immunosuppressive therapy, the first step should be a careful reassessment of the effectiveness of the current regimen in controlling vascular inflammation. If the existing regimen is insufficient for LVV, escalation to a conventional immunosuppressive strategy is recommended. For GCA, this typically involves a glucocorticoid-based regimen combined with methotrexate. For TAK, glucocorticoids in combination with methotrexate, azathioprine, or TNF inhibitors should be considered. Conversely, if the ongoing immunosuppressive regimen is effective in controlling LVV, TNF inhibitors are suggested as first-line agents to maintain disease control. In cases of inadequate response or intolerance, newer targeted therapies, including monoclonal antibodies against IL-17 or IL-23 may be considered as second- or third-line treatment options (17).

In addition to infliximab, successful treatment of concomitant LVV and IBD have been reported with ustekinumab, JAK inhibitors, and tocilizumab. One report described a 17-year-old girl with concomitant TAK and CD, confirmed by PET-CT and colonoscopy, who was positive for HLA-B52. Initial glucocorticoid therapy was ineffective, whereas subsequent treatment with ustekinumab in combination with 5-aminosalicylic acid enabled successful glucocorticoid withdrawal and sustained remission of both conditions (59). Given the shared immunopathological mechanisms of TAK and CD, particularly their association with IL12B gene polymorphisms, ustekinumab, which targets the IL-12/IL-23 p40 subunit, represents a biologically plausible therapeutic option. Janus kinase inhibition has also shown promise. A patient with TAK-associated UC who failed conventional therapy, golimumab, and vedolizumab was treated with tofacitinib, resulting in rapid clinical improvement, normalization of inflammatory markers, regression of vascular wall thickening, and eventual endoscopic remission (60). Experience with tocilizumab, an interleukin 6 receptor antagonist, has been mixed. A Japanese case reported a patient with UC complicated by TAK and bronchial artery–derived hemoptysis who responded favorably to prednisolone combined with tocilizumab. After two months of therapy, prednisolone was successfully tapered, and no further systemic, respiratory, or gastrointestinal symptoms were observed (61, 62). In contrast, another reported case described a patient with TAK and UC in whom tocilizumab failed to control disease activity and instead appeared to exacerbate intestinal inflammation, potentially increasing the risk of intestinal perforation. This adverse effect may be explained by the dual role of IL-6 in intestinal mucosal protection and regeneration, whereby IL-6 blockade could impair intestinal mucosal repair processes (62). Similarly, a 14-year-old boy with CD who developed TAK during anti-TNF therapy experienced worsening intestinal symptoms after switching from infliximab to tocilizumab, despite improvement in vascular inflammation (63). Currently, evidence supporting the use of tocilizumab in IBD remains limited (64). Consequently, it should be used with particular caution in patients with LVV complicated by IBD. Achieving simultaneous and durable control of both diseases remains challenging, underscoring the need for individualized biologic selection based on disease phenotype, prior treatment response, and risk–benefit assessment.

In summary, the management of IBD complicated by LVV requires a comprehensive assessment of disease activity and therapeutic response in both the intestinal and vascular systems. Treatment selection and combination strategies should be individualized to achieve balanced control of inflammation across affected organs, to maximize efficacy while minimizing the risks of relapse and complications (**Figure 3**). A summary of the cases of LVV combined with IBD discussed in this article is presented in **Table 1**.

**Table 1:** Summary of cases of LVV combined with IBD.

No. (references)	Predominant phenotype	Number of cases	Treatment	Outcome
1 (22)	TAK+CD/(IBDU)	9	anti-TNFα	Improved
2 (40)	TAK+CD	1	Infliximab+azathioprine+anticoagulation	Improved
3 (56)	TAK+CD	2	anti-TNFα	Improved, serious infection in one
4 (31)	TAK+CD	1	prednisone +methotrexate	Improved
5 (57)	TAK+UC	1	anti-TNFα	TAK progression
6 (59)	TAK+CD	1	ustekinumab+5-aminosalicylic acid	Improved
7 (60)	TAK+UC	1	tofacitinib	Improved
8 (61)	TAK+UC	1	tocilizumab	Improved
9 (62)	TAK+UC	1	tocilizumab	Worsened
10 (63)	TAK+CD	1	tocilizumab	Worsened

## Conclusion

Although uncommon, the coexistence of LVV and IBD is increasingly recognized as a clinically meaningful association. The two conditions show substantial overlap in epidemiologic patterns, immunogenetic background, and pathogenic mechanisms, with the strongest association observed between TAK and CD. Shared HLA susceptibility loci, Th1- and Th17-driven immune responses, and proinflammatory cytokine networks appear to constitute key biological links between intestinal and vascular inflammation. Recent evidence has also highlighted the potential role of monogenic immune dysregulation in severe, treatment-refractory cases involving both intestinal and vascular inflammation, suggesting that targeted interventions such as allogeneic-HSCT may offer curative potential in selected patients. Certain biologic agents, particularly TNF-α inhibitors, may exert dual and sometimes paradoxical effects, contributing to disease control in some patients while inducing or exacerbating vasculitis in others. This complexity underscores the need to carefully balance therapeutic benefits and risks. Accurate diagnosis and optimal management of LVV-IBD require integrated imaging, endoscopic assessment, and close multidisciplinary collaboration to facilitate early recognition and reduce the risk of complications. Future multicenter, large-scale prospective studies are warranted to better define disease phenotypes, elucidate the natural history of this comorbidity, and establish evidence-based treatment strategies. Improved understanding of shared immune pathways may enable more precise, targeted interventions, ultimately enhancing long-term outcomes.
